# *ATP8B1* Gene Expression Is Driven by a Housekeeping-Like Promoter Independent of Bile Acids and Farnesoid X Receptor

**DOI:** 10.1371/journal.pone.0051650

**Published:** 2012-12-10

**Authors:** Dita Cebecauerová, Sandra S. Strautnieks, Jane A. Byrne, Milan Jirsa, Richard J. Thompson

**Affiliations:** 1 Institute of Liver Studies, King's College London School of Medicine, at King's College Hospital, London, United Kingdom; 2 Laboratory of Experimental Hepatology, Institute for Clinical and Experimental Medicine, Prague, Czech Republic; University Hospital S. Maria della Misericordia, Italy

## Abstract

**Background:**

Mutations in *ATP8B1* gene were identified as a cause of low γ-glutamyltranspeptidase cholestasis with variable phenotype, ranging from Progressive Familial Intrahepatic Cholestasis to Benign Recurrent Intrahepatic Cholestasis. However, only the coding region of *ATP8B1* has been described. The aim of this research was to explore the regulatory regions, promoter and 5′untranslated region, of the *ATP8B1* gene.

**Methodology/Principal Findings:**

5′Rapid Amplification of cDNA Ends using human liver and intestinal tissue was performed to identify the presence of 5′ untranslated exons. Expression levels of *ATP8B1* transcripts were determined by quantitative reverse-transcription PCR and compared with the non-variable part of *ATP8B1*. Three putative promoters were examined *in vitro* using a reporter gene assay and the main promoter was stimulated with chenodeoxycholic acid. Four novel untranslated exons located up to 71 kb upstream of the previously published exon 1 and twelve different splicing variants were found both in the liver and the intestine. Multiple transcription start sites were identified within exon −3 and the proximal promoter upstream of this transcription start site cluster was proven to be an essential regulatory element responsible for 70% of total *ATP8B1* transcriptional activity. *In vitro* analysis demonstrated that the main promoter drives constitutive *ATP8B1* gene expression independent of bile acids.

**Conclusions/Significance:**

The structure of the *ATP8B1* gene is complex and the previously published transcription start site is not significant. The basal expression of *ATP8B1* is driven by a housekeeping-like promoter located 71 kb upstream of the first protein coding exon.

## Introduction

Mutations in *ATP8B1* (18q21-q22) cause variable cholestatic phenotypes ranging from progressive to benign recurrent forms (Progressive Familial Intrahepatic Cholestasis type 1, PFIC1, formerly Byler disease, and Benign Recurrent Intrahepatic Cholestasis type 1, BRIC1; OMIM 211600, 243300) [1], [2]. ATP8B1 deficient patients suffer from intrahepatic cholestasis, often accompanied with extrahepatic symptoms including diarrhoea, pancreatitis and hearing problems. Milder phenotype presents with recurrent attacks of cholestasis typically without permanent liver damage [3]. Serum γ-glutamyltranspeptidase (γ-GT) activity and cholesterol concentrations are normal.

The ATP8B1/FIC1 (Familial Intrahepatic Cholestasis 1) protein, a member of the P4-type ATPases subfamily, is widely expressed in epithelial tissues [4], [5], [6] and is considered a phosphatidylserine flippase, translocating phosphatidylserine from the outer to the inner leaflet of the plasma membrane [6], [7]. The ATP8B1 disease mechanism is, however, poorly understood. *In vivo* experiments using “Byler“ Atp8b1^G308V/G308V^ mice or ATP8B1 deficient hepatocytes demonstrated defective membrane order due to the impaired flippase activity of ATP8B1 [8], [9]. A more recent study [10] challenged the proposed mechanism and on ATP8B1-depleted Caco-2 cells demonstrated an unimpaired flippase activity, with profound disorganization of apical actin cytoskeleton and loss in microvilli. Since ATP8B1 deficiency is primarily characterised by cholestasis, some studies attempted to attribute the phenotype to a defective farnesoid X receptor (FXR) signalling pathway [11], [12], [13]. Others [14] suggested that impaired FXR activity is secondary to cholestasis and, as such, is not responsible for the PFIC1/BRIC1 phenotype. Unperturbed activity of FXR and its target genes was observed in ATP8B1-depleted Caco-2 cells using small hairpin RNA and small interfering RNA respectively [9], [10], which suggests an unimpaired FXR signalling pathway in PFIC1/BRIC1 patients.

In a large study by Klomp et al. [15] *ATP8B1* mutations were detected in fewer than 50% of the families screened, irrespective of severity of their disease. It may be partly because only the coding region of the gene was analyzed. Even though the *ATP8B1* gene is important biologically and clinically, our knowledge of its regulatory regions remains limited. Our aim was to characterise the transcriptional control of the *ATP8B1* gene by identifying its promoter and 5′untranslated (5′UTR) regions, and to search for putative regulatory sites in any newly discovered parts of the gene.

## Results

### The 5′UTR of ATP8B1 Comprises Four Novel Exons and Spans a 71 kb Genomic Region

**Figure 1:**
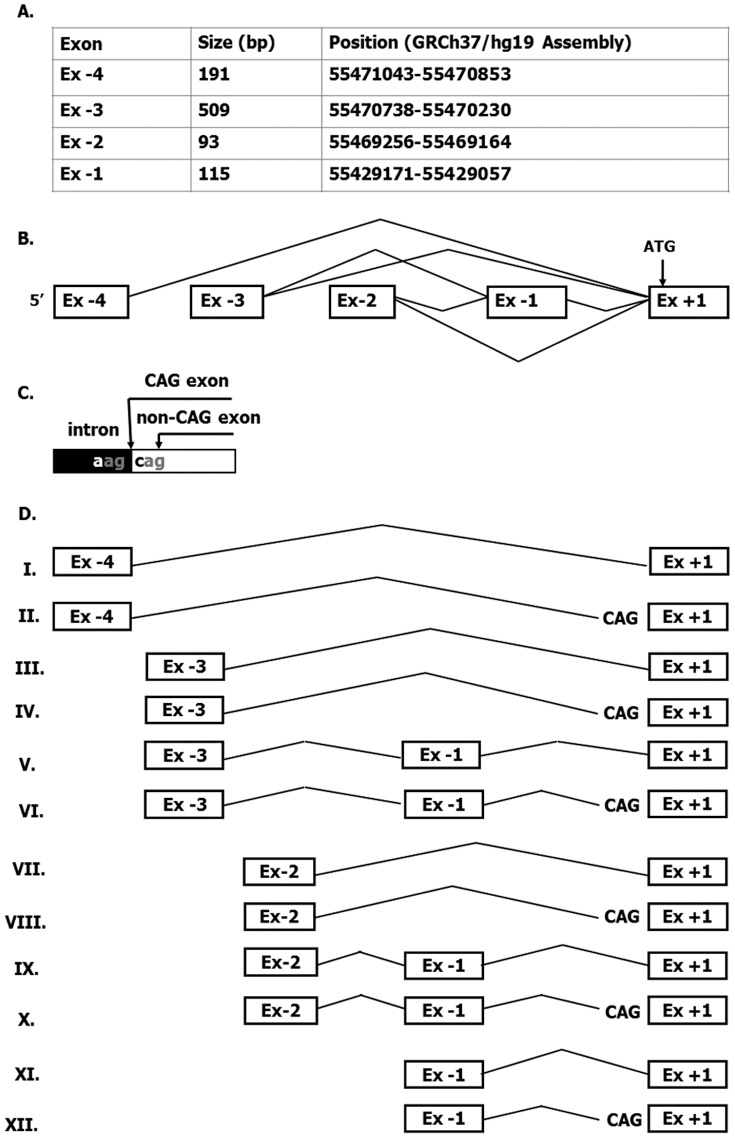
The heterogeneity of 5′UTR of *ATP8B1* gene. (A) Length and position of novel untranslated exons of *ATP8B1* gene. (B) Six identified alternatively spliced variants are indicated by diagonal lines. (C) The existence of two acceptor splice sites CAGCAG (tandem acceptors) at the 5′ boundary of the first translated exon (Ex +1) of *ATP8B1* allows the generation of two different splice forms for each combination of upstream exons with Ex +1. Thus generated splice forms differ from each other by only three nucleotides CAG and give rise to twelve *ATP8B1* isoforms in total (D).

5′RACE using RNA from a number of different human tissues including liver, small intestine, large intestine and pancreas, revealed four novel untranslated exons located 30, 70, 71 and 72 kb upstream of the known exon +1 (Ex +1). These new exons have been designated exons −1 to −4 (Ex −1 to Ex −4) and their lengths and positions are summarized in Fig. 1A. Six different splicing variants comprising different combinations of the novel untranslated exons were detected (Fig. 1B). Due to the existence of two donor splice sites (tandem acceptors) at the 5′end of Ex +1, two different ways of splicing the untranslated exons to Ex +1 are possible (Fig. 1C). Indeed, two different variants for each splicing event including Ex +1, differing from each other by only 3 bases (CAG), were observed. This subtle change represents a further source of 5′UTR variability which generates, in total, twelve *ATP8B1* mRNA isoforms (Fig. 1D).

**Figure 2:**
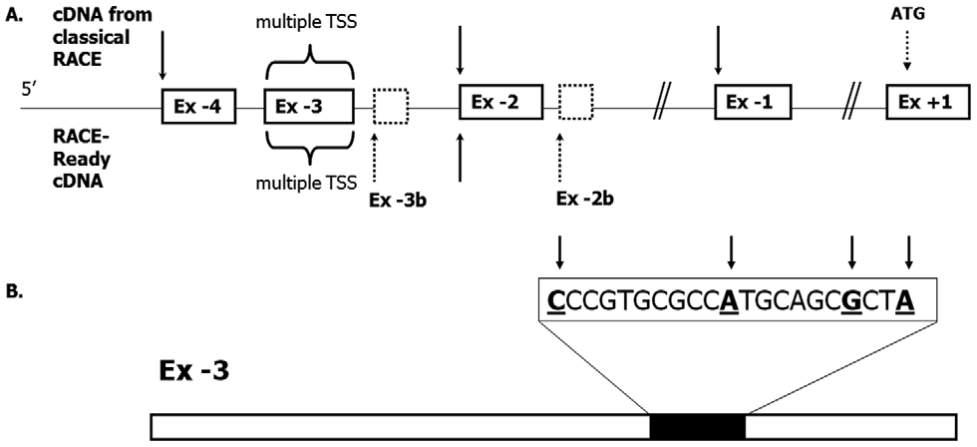
Transcription start sites (TSSs) identified within 5′UTR of *ATP8B1* gene. (A) Transcription of *ATP8B1* gene originates from multiple TSSs (indicated by arrows); two additional TSS were identified at the beginning of rarely used exons, adjacent to Ex −3 and −2 respectively (dashed boxes). Sixteen different TSSs were found within the 509 bp-long Ex −3 with the main cluster located between nucleotides 135 and 115 upstream from the 3′end of Ex −3 (B).

Using commercially available First Choice® RACE-Ready cDNA from human liver and intestinal tissue, several putative transcription start sites were identified: One each at the beginning of the novel UTR exons depicted in Fig. 2A, except Ex −1, and several alternative transcription start sites within Ex −3. A transcription start site cluster was located in the region between nucleotides 135 and 115 upstream from the 3′end of Ex −3 (Fig. 2B). This region contains a putative initiator sequence (*Inr*) [16], [17]. RACE-Ready experiments also identified two other novel exons in the vicinity of Ex −3 and Ex −2, designated Ex-3b (Chr18: 55470138-55470074; GRCh37/hg19) and Ex −2b (Chr 18: 55468948-55468914; GRCh37/hg19) (Fig. 2A). Ex −4, which was identified by classical RACE above, was not detected using RACE-Ready cDNA. The transcription start site (TSS) at the beginning of Ex −1 was only found by RACE, while using RACE-Ready cDNA Ex −1 was a part of transcripts initiating further upstream of Ex −1.

### Consistent Expression Pattern of the Novel ATP8B1 mRNA Isoforms in the Liver and Intestine

**Figure 3:**
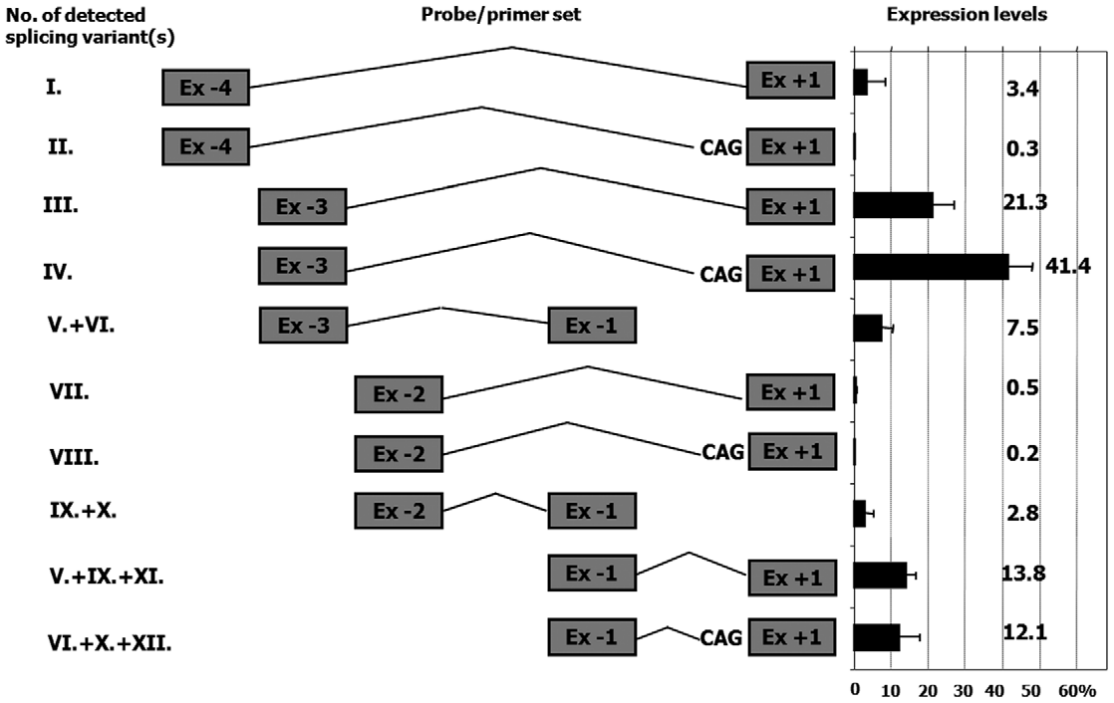
Relative expression levels of different splicing forms assessed by qRT-PCR. Diagram of individually designed probes (for probe and primer sequences see Table S1) used to evaluate the expression levels of twelve identified splicing variants of *ATP8B1* 5′UTR. Tested splicing variants are indicated by Latin numbers on the left, average expression levels for each transcript from normal liver tissues (n = 7) are shown on the right. The expression levels are presented as a relative value normalised to the expression of the protein coding region represented by Ex +1/Ex +2 boundary.

To define the biological relevance of the different *ATP8B1* transcripts we used 7 normal human livers and 3 normal human intestinal samples in qRT-PCR experiments with individually-designed probe/primer sets covering all identified alternative exon-exon boundaries found by 5′RACE (Tab. S1). The results are presented in Fig. 3, where the diagrams represent probe/primer sets and Latin numbers relevant splicing variant(s) detected. The results show the abundance of each 5′UTR splicing variant relative to the protein coding region, represented by the Ex +1/+2 boundary. qRT-PCR demonstrated the prevalent expression of transcripts containing Ex −3 directly spliced to Ex +1; these splicing variants, Ex −3/Ex +1 and Ex −3/CAG/Ex +1 (Fig. 3) comprise almost 2/3 of total *ATP8B1* expression, whilst the alternative splicing event, Ex −3/Ex −1 that in fact comprises two mRNA isoforms: Ex−3/Ex −1/Ex +1 and Ex−3/Ex −1/CAG/Ex +1, comprises less than 10% of the total transcripts. Splicing variants Ex −1/Ex +1 and Ex −1/CAG/Ex +1 account for almost 26% but splicing events including Ex −2 account for less than 4% of the total transcripts. The expression level of Ex −4/Ex +1 (Fig. 3, variants I and II), found by classical 5′RACE but not RACE-Ready cDNA, varied significantly amongst the samples, ranging from complete absence to 3.4% of total gene expression. The expression levels of the two small rarely detected exons, located 91 bp and 215 bp downstream from Ex −3 and Ex −2 respectively (Ex−3b and Ex −2b, Fig. 2A), and identified only using RACE-ready cDNA, were below 1% in all experiments (data not shown). Additional qRT-PCR experiments on a limited (n = 3) number of human intestinal samples did not exhibit any significant difference compared to the expression pattern detected in liver, with Ex −3 proving to be the most prevalent 5′UTR exon expressed in both, liver and intestinal tissues (data not shown).

Searching of the NCBI EST database (National Centre for Biotechnology and Information, http://www.ncbi.nlm.nih.gov/) revealed one *ATP8B1* transcript with Ex −3 spliced to Ex +1 without the CAG triplet (GenBank accession: DR005588.1, GRCh37/hg19). This transcript does not include the protein coding Ex +2, and thus Ex +1 must be spliced directly to Ex +3. The resultant predicted protein sequence would have a premature stop codon (TAA) at position 62, which would not give a viable ATP8B1 protein. We searched for the existence of Ex +1/Ex +3 splicing event by PCR using cDNA templates prepared from liver and intestinal RNA. qRT-PCR using the specifically-designed probe for the Ex +1/Ex +3 boundary demonstrated 20-fold lower expression of this transcript in normal human liver and intestinal tissue compared to the Ex +1/Ex +2 splicing variant (data not shown). The biological significance of the low abundance transcript therefore remains unclear.

Also, our results did not confirm the existence of previously identified 909 bp-long *ATP8B1* 5′exon [15].

### In Silico Identification of the Putative Promoters in ATP8B1 Gene

**Figure 4:**
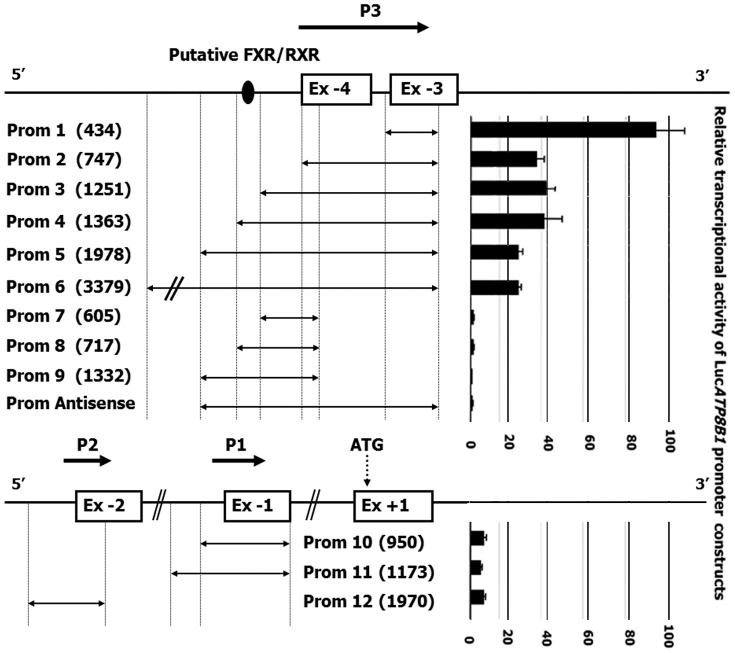
Functional analysis of *ATP8B1* promoter regions. HepG2 cells were transiently transfected with luciferase reporter gene constructs containing 12 different fragments of putative *ATP8B1* promoters. Nine luciferase constructs (Prom 1 to Prom 9) were designed to comprise the putative dominant promoter P3, two constructs covered promoter P1 (Proms 11 and 12) and one covered promoter P2 (Prom 10). The position of the tested fragments are indicated by horizontal double arrow lines. The number in brackets next to the construct name represents its size (bp). Prom 3 and Prom 4 were designed to include/exclude a putative FXR/RXR binding site indicated by black oval. Antisense construct encodes the same region as Prom 5, but in antisense orientation. Putative promoters (P1–P3) are depicted as horizontal thick arrows. Transcriptional activity for each construct was measured in relative light units per second (RLU/s) and corrected for the transfection efficiency using the internal control *Renilla* pRL-TK expression plasmid. The data shown are calculated from 3–5 independent experiments and related to the pGL3 Basic activity.

On the basis of the 5′RACE and qRT-PCR results, we predicted the major promoter region of *ATP8B1* to be located upstream of the cluster of TSSs within Ex −3 (P3, Fig. 4) and two weaker promoters to be upstream of Ex −2 and −1 (P2 and P1, Fig. 4). In agreement with our hypothesis, *in silico* analysis employing three independent promoter prediction programs located putative promoter regions within a CpG island 70–72 kb upstream of the protein coding Ex +1 corresponding to the chromosomal location of the novel Ex −3 (Fig. S1). Computer analysis did not predict the presence of promoter upstream of Ex −1.

### Experimental Validation of the Major Promoter of the ATP8B1 Gene

In order to identify whether the major promoter of *ATP8B1* resides upstream of Ex −3, twelve promoter constructs (Prom 1–12, Fig. 4) utilising the luciferase reporter gene system were prepared: Six comprised the sequences upstream of the TSS cluster in Ex −3, whilst three focused on the region upstream of Ex −4 and three represented the regions upstream of Ex −2 and Ex −1. The promoter sequences cloned ranged from 434 bp to 3379 bp in length.

The luciferase assay results, summarised in graph on Fig. 4, demonstrate the highest relative promoter activity for the short 434 bp construct (Prom 1) which is situated immediately upstream of the major TSS cluster in Ex −3. Assessment of the longer promoter constructs ranging from 747 bp up to 3379 bp (Proms 2–6) exhibited approximately 65% reduced promoter activity compared to the Prom 1 construct. Only minimal differences in the reporter assay were observed among Proms 2–6 constructs relative to each other. Removal of the 434 bp region upstream of Ex −3 resulted in a complete absence of luciferase activity in all constructs tested (Proms 7–9; Fig. 4), thus emphasizing the importance of the proximal P3 promoter in driving luciferase activity.

A putative TSS in Ex-1, not predicted *in silico*, was detected only by classical 5′RACE. Nevertheless, the 40 kb distance between the main promoter and Ex −1 in combination with the qRT-PCR results suggested the presence of an alternative regulatory region in the vicinity of Ex −1. Constructs, which included the putative promoter upstream of Ex −1 and also upstream of Ex −2 (Proms 10–12), showed only basal transcription activity which was less than 10% that of the Prom 1 construct upstream of Ex −3 (Fig. 4).

### The 5′UTR of ATP8B1 is Highly Conserved between Human, Mouse and Rat

**Figure 5:**
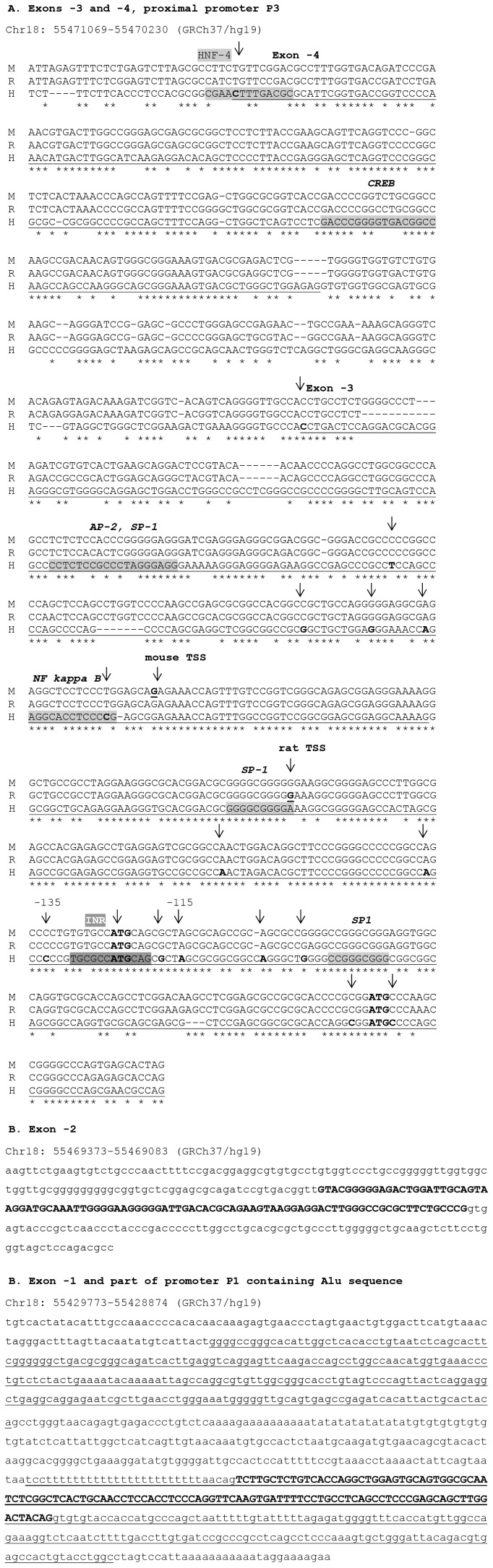
Putative transcription factor binding sites and conservation of *ATP8B1* 5′UTR. (A) Sequence alignment using ClustalW algorithm (http://www.ebi.ac.uk/Tools/clustalw/). High level of conservation among mouse (M), rat (R) and human (H) genome was detected for Ex −3 and Ex −4 (conserved nucleotides indicated by stars). Putative Sp-1, Ap-2, NFκB transcription factor binding sites were predicted in exonic/promoter region P3. Putative CREB and HNF-4 binding sites were identified within a distal part of promoter P3 corresponding to Ex −4 sequence. Initiator element sequence encompasses the main TSS cluster in Ex −3. Exonic regions are underlined, transcription start sites are indicated by arrows and bold letters and putative transcription factor binding sites by grey boxes. Two upstream ATG are in bold. (B) Sequence of Ex −2 and (C) Ex −1. Exonic region is highlighted in bold. Alu consensus sequences are underlined.

The sequences of mouse and rat *Atp8b1* 5′UTR were obtained from Ensembl database (http://www.ensembl.org/index.html) and aligned with nucleotide sequences of newly identified human UTR exons using the ClustalW (http://www.ebi.ac.uk/clustalw) program. A high degree of conservation was found in the region corresponding to Ex −3 and Ex −4: 83% and 82%, respectively, for a human-mouse alignment and 66% and 69%, respectively, for a human-rat alignment (Fig. 5).

### Transcription Factor Binding Sites Present in the ATP8B1 Promoter Region

No consensus TATA or CAAT boxes were found in the proximal P3 promoter of the *ATP8B1* gene. On the other hand, several putative binding sites for non-specific, general transcription factors (Sp1, AP-2, NFκB) were identified in that DNA sequence. No liver- or intestine-specific transcription factor binding sites were found in the vicinity of the TSS cluster of Ex −3 (Fig. 5). A putative FXR binding site was identified using only the MatInspector computer analysis software, and this was 807 bp upstream of Ex −4. However, this site, **GAGT**G**A**c**TGACC**A, does not correspond to any known consensus FXR binding sequence and the sequence is not conserved between human, mouse and rat.

### Influence of Bile Acids on Promoter P3 Activity

To investigate the effect of bile acids on P3 promoter activity, HepG2 cells were transfected with the Prom 3, Prom 4, and Prom 6 *ATP8B1* luciferase constructs, which comprise the main promoter P3, and then incubated in the presence or absence of CDCA and TCA for 24 hours. Prior to the commencement of the luciferase experiments, we evaluated the expression levels of endogenous *ATP8B1, ABCB11, SHP* and *CYP7A1* by qRT-PCR before and after CDCA treatment to assess the normal response of HepG2 cells to bile acid stimulation. While *ATP8B1* levels remained constant, *ABCB11* and *SHP* mRNAs were up-regulated and *CYP7A1* mRNA down-regulated in the presence of 50 and 100 µM CDCA respectively (data not shown), proving HepG2 as a suitable model to assess the response of the *ATP8B1* promoter constructs to bile acids.

**Figure 6:**
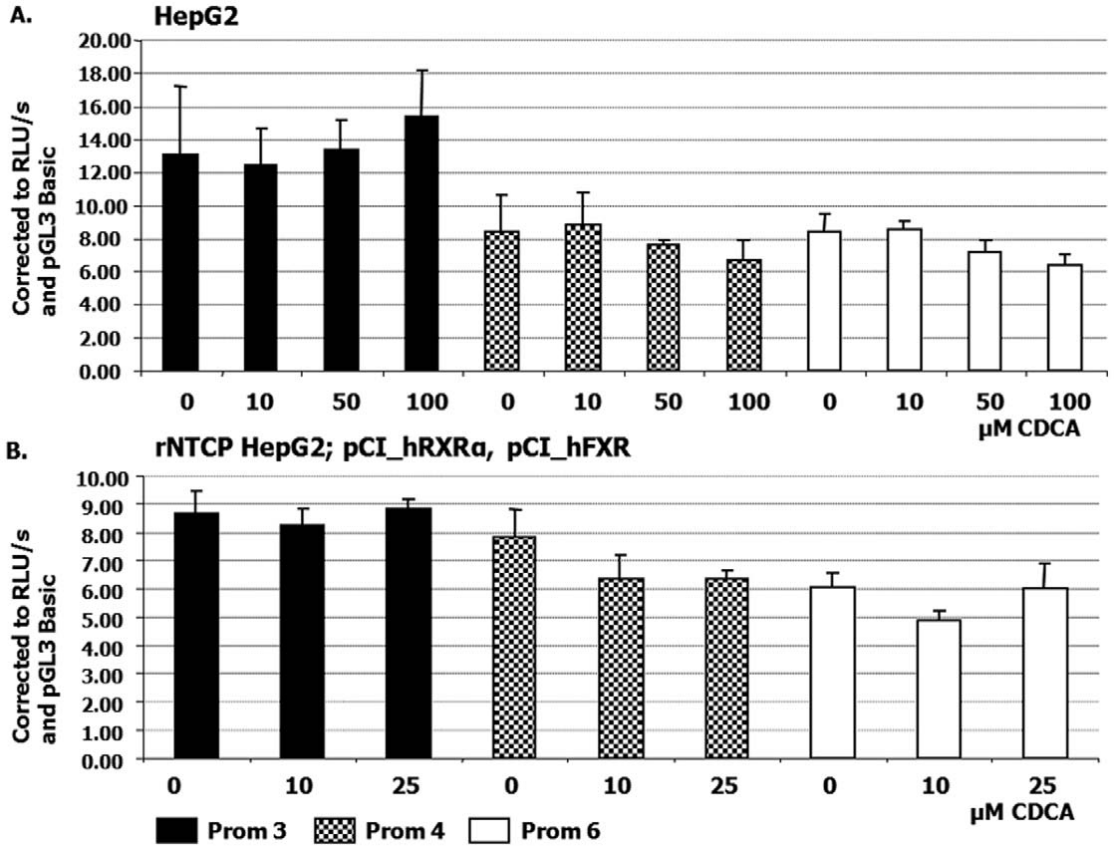
*ATP8B1* promoter activity in cells stimulated with bile acids. No significant change in activity was detected after stimulation with CDCA. (A) HepG2 cells were transiently transfected with three previously characterised (Fig. 4) *ATP8B1* promoter gene constructs (Prom 3, 4 and 6) and stimulated with 0, 10, 50 and 100 µM CDCA for 24 hours. All constructs comprise proximal 434 bp-promoter P3, Prom 4 includes putative FXR binding site identified by MatInspector computer analysis software, and Prom 6 represents the largest construct containing 3379 bp of 5′flanking region. (B) HepG2 cells stably expressing rat sodium-taurocholate co-transporting polypeptide (rNtcp) were transiently transfected with constructs Prom 3, 4 and 6 together with 50 ng of pCI_hRXRα and 50 ng pCI_hFXR plasmids and treated with 0, 10 and 25 µM CDCA for 24 hours.

In agreement with the unchanged mRNA expression of *ATP8B1* under CDCA stimulation, none of the luciferase constructs tested demonstrated a significant change in promoter activity in HepG2 cells after CDCA (Fig. 6A) or TCA (not shown) treatment. Since HepG2 cells do not express the NTCP, all experiments were repeated in a HepG2 cell line stably expressing rat Ntcp (rNtcp-HepG2 cells), that were in addition co-transfected with vectors expressing human FXR and RXRα. To minimise the effect of bile acids present in fetal calf serum, the cells were cultured in medium containing charcoal-stripped fetal bovine serum with minimal content of bile acids [13]. In agreement with the previous experiments, no significant change in *ATP8B1* promoter activity was observed (Fig. 6B). These results indicate no direct link between FXR, bile acids and the *ATP8B1* major promoter.

## Discussion

In this study we demonstrate that *ATP8B1* expression is regulated by a highly structured 5′UTR which spans 71,964 kb and comprises four untranslated exons located a considerable distance upstream of the first protein coding exon. These exons are alternatively spliced. The main TSS cluster was located within nucleotides 135 to 115 upstream from the 3′end of Ex −3. This TSS cluster contains a putative initiator element (*Inr*) [17], [18] and is encompassed by Sp1 binding sites (Fig. 5). The previously published TSS [1], [15] is not significant. Transcription of *ATP8B1* is driven by three newly identified promoters (P1, P2 and P3; Fig. 4) In liver, the promoters P1 and P2 play only minor role under physiological conditions. Promoter P3 was identified as the essential regulatory element responsible for 70% of total *ATP8B1* gene expression. The 434 bp part of P3 (Prom 1 construct in Fig. 4) upstream of the main TSS cluster promiscuously serves both as exonic and promoter region and represents the main driving force of *ATP8B1* gene expression. The importance of this region was further confirmed by sporadic use of TSSs located further upstream of the main TSS cluster in Ex −3.

The dominant *ATP8B1* promoter P3 displays typical features for promoters of housekeeping genes: TATA-less, GC-rich sequence with multiple TSSs [19] in which only non-specific putative transcription factor binding sites for Sp1, AP-2 and NFκB were identified. (Fig. 5). These data are in agreement with the ubiquitous expression of ATP8B1 (FIC1) and its putative complex role in maintenance of apical membrane structure [8], [10].

Genes regulated by alternative promoters are common in humans. Multiple promoters can be utilised according to environmental conditions or to a particular developmental stage to ensure the tissues-specific or spatio-temporal expression of the appropriate gene isoform [20]. Various mRNA isoforms may also interact to achieve a transcriptional repression of an alternative transcript [20], [21]. It has been shown that of several alternative mRNA isoforms, one can be ubiquitously expressed among various cell types, whereas the remaining ones may be limited to a small number of tissues [22], [23], [24], [25]. This might be the case of *ATP8B1* alternative transcripts. Even though our study does not support such tissue specific regulation of *ATP8B1* at the transcriptional level in the tested samples, further research could characterise the role of all three promoters in different organs under varying conditions and address the involvement of post-transcriptional control mechanisms.

In a view of the complex structure of the *ATP8B1* gene and its highly variable mRNA levels across cell types, RNA stability and post-transcriptional control appears to be more important in *ATP8B1* regulation than previously expected. Our data demonstrate significant differences between the activity observed for the promoter upstream of Ex −1 (P1) versus the promoter upstream of Ex −3 (P3). However, this difference was not replicated at the level of abundance of the mRNA transcripts associated with these promoters: Whereas the reporter gene activity mediated by promoter P1 was 15-fold lower than that of the principle promoter P3 (Fig. 4), the mRNA levels of corresponding transcripts displayed only a 3-fold difference (Fig. 3). The observed discrepancy suggests possible differential efficiencies in post-transcriptional processing of the corresponding pre-mRNA *ATP8B1* forms.

5′UTRs are known as key mediators of post-transcriptional control. The mechanisms of UTR-mediated regulation comprise, among others, stable secondary structures including those formed by repetitive sequences such as Alu and upstream open reading frames [26]. Alternatively spliced Ex −1 of *ATP8B1* is apparently an exonized Alu [27] element with the promoter P1 containing complementary Alu sequence. Alu sequences embedded in 5′UTR have been shown to modulate both transcription and translation [28], [29].

**Figure 7:**
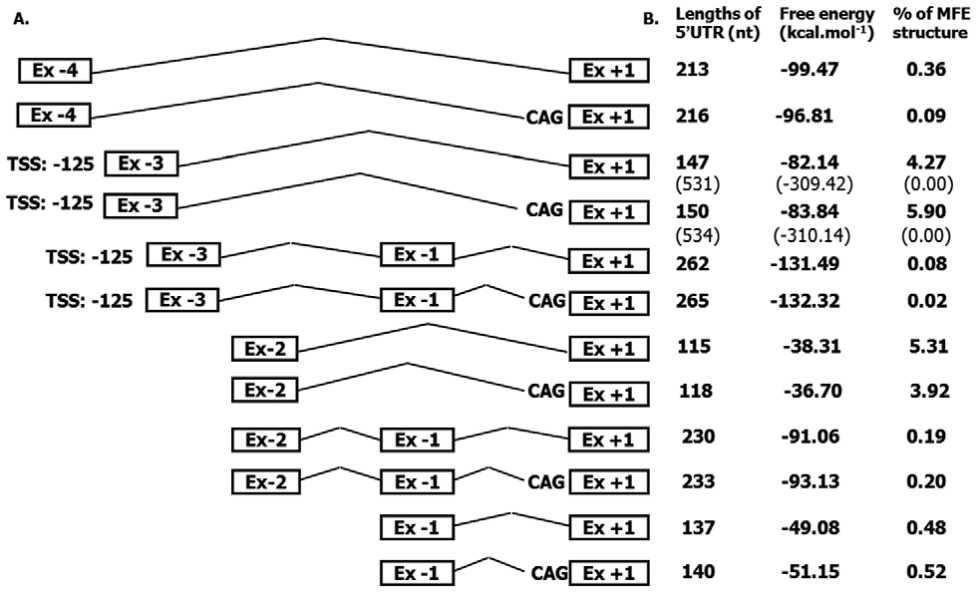
Thermodynamic properties of identified 5′UTR isoforms. The comparison of the 5′UTR length and RNA secondary structure free energy and percentage of minimal free energy (MFE) for all identified *ATP8B1* 5′UTR isoforms, schematically depicted on the left. Prediction for the most frequent isoforms initiating at Ex −3 was calculated using TSS at position −125 from 3′end of Ex −3. Data in brackets (row 3 and 4) represent data for TSS at position −509. Putative secondary RNA structures predicted using RNAfold web tool (http://rna.tbi.univie.ac.at/cgi-bin/RNAfold.cgi) are summarised in Fig. S2.

Other potent modulators of transcriptional and translational efficiency are Upstream Open Reading Frames (uORFs) which can affect gene expression by inhibition of mRNA stability and translational repression [30], [31]. Recently demonstrated uORF-mediated ability to trigger the nonsense-mediated mRNA decay [32] ^,^ [33] or to inhibit the downstream ORF by upstream located uAUG [34] represents processes that could be potentially involved in posttranscriptional regulation of *ATP8B1*. Indeed we identified *ATP8B1* transcripts that differ in their leader sequences and in the presence of putative upstream start codons AUG (uAUG) (Fig. 5). Whereas no uAUG was found within the transcript containing Ex −1, two uAUGs and two uORFs were identified within the prevalent transcript containing Ex −3. Further factors known to influence regulation of gene expression [35], [36], [37] are heterogeneity, a high GC content and the unusually long length of 5′UTR. Their potential contribution to regulation of *ATP8B1* expression is discussed in Fig. 7, Fig. S2 and their legends.

Since *ATP8B1* deficiency is associated with cholestatic liver disease, most studies have focused on the role of the gene in cholestasis. Zollner and colleagues [38] found no changes in *ATP8B1* mRNA level in cholestatic patients. In our experiments, stimulation of HepG2 cells with CDCA or TCA respectively showed no change in *ATP8B1* mRNA levels and CDCA or TCA treatment of HepG2 or rNTCP-HepG2 cells expressing various *ATP8B1* promoter constructs did not significantly alter the luciferase activity compared with untreated cells. This observation is in agreement with the gene’s ubiquitous expression and suggests that bile acid independent mechanisms regulate ATP8B1 expression across different cell types.

In conclusion our study provides fundamental data about the complexity of *ATP8B1* gene regulation. Newly identified *ATP8B1* mRNA isoforms differ in their 5′UTRs and both transcriptional and post-transcriptional efficiency. The basal expression of *ATP8B1* gene in the liver and the intestine is driven by a promoter with house-keeping like properties. Regulatory parts of *ATP8B1* characterised in this study represent a feasible region for mutational search in patients with features suggestive of ATP8B1 deficiency, in whom no mutations have been identified within the coding region.

## Materials and Methods

### 5′Rapid Amplification of cDNA Ends (5′RACE)

The 5′ends of the *ATP8B1* gene were mapped using the 5′/3′ RACE kit, 2^nd^ Generation (Roche, Switzerland) according to manufacturer’s instructions. Total RNA was isolated using RNA-Bee (Tel-test, Inc., Friendswood, USA) from 50 mg of human liver or intestinal tissue, or 5×10^6^ of HepG2 cells. All procedures were conducted with written informed consent under an institutional-review-board approved protocol or using anonymised bank samples, previously collected with consent for research. To confirm any newly identified transcriptional origins, liver and intestinal RACE-ready cDNA (Ambion, Austin, USA) was used. *ATP8B1* gene-specific primers for RACE were designed to span the junctions of exons 3/4, 2/3 and 1/2. Resultant PCR products were cloned into the pDrive Cloning Vector (Qiagen, Hilden, Germany) and sequenced in both directions using ABI Big Dye (Version 3.1) on a 3100 automated DNA Sequencer (Applied Biosystems, Foster City, USA) using vector specific primers.

### Quantitative Real–time PCR

Twelve sets of individually designed TaqMan® MGB probes labelled with Fam and non-fluorescent quencher and primers were generated using Primer Express® Software Version 2.0 (Applied Biosytems, Warrington, UK), to cover all variants of alternative splicing of the untranslated exons (Table S1, No.1-12). 100 ng of DNase-treated total RNA from normal human liver and intestinal samples was used as a template in a 20 µl reverse transcription reaction using Transcriptor (Roche, West Sussex, UK) and mix of random hexamer or gene specific primers (Invitrogen, Paisley, UK, Sigma-Aldrich, Dorset, UK). 1 µl of first strand cDNA was then assessed in triplicate for levels of the different *ATP8B1* transcripts on an ABI Prism 7000 Sequence Detection System (Applied Biosystems). Expression levels of the studied transcripts and the overall expression of the *ATP8B1* coding sequence represented by Ex +1/Ex +2 boundary, were corrected to the level of 18S rRNA (delta Ct) (TaqMan® MGB probe, Applied Biosytems, Warrington, UK). A PCR of non-reverse transcribed RNA was performed as a negative control to check for any genomic DNA contamination. Delta delta Ct values were calculated using ABI SDS software with RQ study application (Version 1.2.3, Applied Biosystems) and the data analysed using Microsoft Excel.

### Promoter and First Exon Analysis in Silico

Three independent algorithms for promoter prediction (http://genome.ucsc.edu/, http://www.genomatix.de, http://bimas.dcrt.nih.gov/molbio/proscan/) were used to analyse the 5′UTR of the *ATP8B1* gene. The University of California Santa Cruz Genome Bioinformatics server was also used to predict the gene’s first exon. The data obtained were compared with the EST database and the 5′RACE experimental results.

### Luciferase and Expression Plasmid Construction

Twelve fragments of the 5′UTR (Prom 1–12 in Fig. 4) of the *ATP8B1* gene were PCR amplified using human genomic DNA as a template, *Pfx* polymerase (Invitrogen) and specific primers containing *Xho*I restriction sites. PCR products were cloned (Invitrogen Zero blunt kit or Qiagen Cloning kit), sequenced, digested with *Xho*I, and ligated into *Xho*I-digested luciferase reporter gene vector pGL3-Basic (Promega, Southampton, UK) using Quick Ligation Kit (New England Biolabs, Hitchin, UK). Resultant constructs were checked for the correct sequence with various restriction enzymes and by direct sequencing prior to transfection. Stocks were prepared using an Endotoxin-free Maxiprep kit (Qiagen, West Sussex, UK). Human retionid X receptor α (hRXRα) and farnesoid X receptor (hFXR) cDNAs were PCR amplified, cloned into Invitrogen’s TOPO TA- cloning kit, sequenced and then sub-cloned to the mammalian expression vector pCI (Promega).

### Cell Culture and Transfection

HepG2 cell lines were purchased from the ATCC (Teddington, UK). Cells were maintained in Dulbecco’s modified Eagle’s medium (PAA, Farnbourough, UK) supplemented with 5% or 10% fetal calf serum (PAA) and glutamine. rNTCP-HepG2 cells, kindly provided by Ulrich Beuers (Department of Gastroenterology and Hepatology, Academic Medical Center, University of Amsterdam, Amsterdam, the Netherlands) and Christopher Rust (Department of Medicine 2– Grosshadern, University of Munich, Munich, Germany), were maintained in Dulbecco’s modified Eagle’s medium containing 5% fetal calf serum and 1 mg/ml G418 (Invitrogen). For transfection, cells were seeded in 24-well plates (TPP) in medium containing 5% fetal calf serum or 5% charcoal-stripped bovine calf serum (GibcoBRL). For transient transfection, 1.5 µl of FuGene HD (Roche) and 500 ng of plasmid DNA were used per well. Plasmid DNA comprised 450 ng of the appropriate Luc*ATP8B1* promoter construct and 50 ng of the *Renilla* pRL-TK internal control plasmid (Promega). For some stimulation experiments, 50 ng of pCI_hFXR and 50 ng of pCI_hRXR contructs were co-transfected with the luciferase constructs. Twenty-four hours after transfection, cells were treated with 0 to 100 µM dimethyl sulfoxide, chenodeoxycholic acid, CDCA or taurocholic acid, TCA (Sigma Aldrich), respectively.

### Reporter Gene Assay

Cells were harvested 48 hours after transfection (24 hours after stimulation with bile acids) and then *Firefly* and *Renilla* luciferase activities in cell lysates were determined using the Dual luciferase system (Promega) on a Glomax luminometer (Promega). All reporter gene assays were performed in triplicate and results are presented as the average value from at least three independent experiments corrected for the transfection efficiency using *Renilla* luciferase activities. The data from individual experiments were related to the activity of the control expression plasmid pGL3 Basic (Promega).

## Supporting Information

Figure S1 ***In silico*** **analysis of first exon and promoter region for** ***ATB8B1*** **gene compared to the 5′RACE results.** Four putative first exons were predicted by “First EF” computer prediction software (horizontal double-arrow lines) [1]. Predicted exons correspond to the chromosomal location of the novel exons −2, −3 and −4 identified in the 5′RACE experiments. Three independent computer algorithms localised putative promoters (dashed horizontal double-arrow lines) in a CpG island 70–72 kb upstream of Ex +1.
(TIF)

Figure S2 **Putative secondary structures of** ***ATP8B1*** **5′UTR isoforms depicted in** **Fig. 7** **of the main text.** Drawings of the minimum free energy (MFE) structures for each splicing variant of *ATP8B1* 5′UTR suggest their possible different role in regulation of gene expression. One of the regulatory mechanism, formation of stable secondary structures, was shown to impede the progress of the scanning ribosome [2]. Such scanning is influenced by the size and the position of the secondary structure(s) towards the 5′cap of the mRNA species: that is, an alternative transcript with a shorter version of the 5′UTR is frequently translated more efficiently than the one with a longer 5′ region [3], [4], [5]. Likewise, a stem-loop structure located a considerable distance from the 5′cap will require a higher free energy compared to one situated closer to it to affect the access of a pre-initiation complex to the mRNA. [2], [6].
(TIF)

Table S1 **Individually designed TaqMan® MGB probes labelled with Fam and non-fluorescent quencher and primers were generated using Primer Express® Software Version 2.0 (Applied Biosytems), to cover all variants of alternative splicing of the untranslated exons.** The abundance of each splicing variant was compared relatively to a non-variable coding region of *ATP8B1* represented by Ex+1/+2 boundary. Probe/primer set for Ex+1/+3 boundary (No.14) was used to test the biological significance of transcript excluding protein coding Ex +2, found in EST database (GenBank accession: DR005588.1). All probe sets were designed across exon/exon boundaries to eliminate the possibility of genomic contamination. Primers used in various amplifications are indicated by upper index (^a, b, c^). The amplification efficiency was tested for each probe/primer set on control templates (obtained by cloning the appropriate cDNA region) using different concentrations of positive and negative controls. As each probe set worked with a slightly different efficiency, the concentration of probes was adjusted for each positive control to reach a cycle threshold (Ct) value difference not greater than 1.
(DOC)

Text S1 **Supporting references.**
(DOC)
