# Supplementary figures and images for: ATP8B1 Gene Expression Is Driven by a Housekeeping-Like Promoter Independent of Bile Acids and Farnesoid X Receptor
Source: PLoS One. 2012 Dec 10;7(12):e51650. doi: 10.1371/journal.pone.0051650 (PMC3518472; doi:10.1371/journal.pone.0051650)

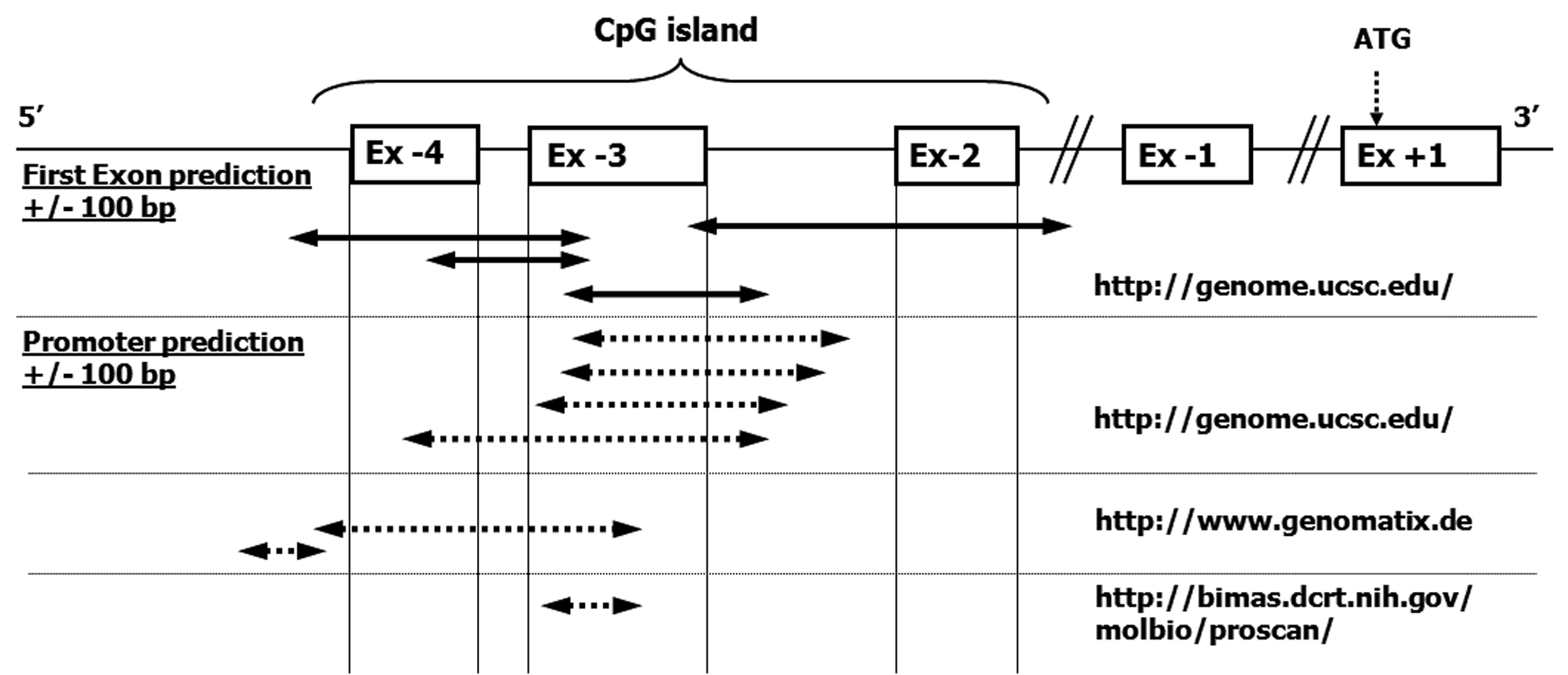

Supplement: Figure S1 — In silico analysis of first exon and promoter region for ATB8B1 gene compared to the 5′RACE results. Four putative first exons were predicted by “First EF” computer prediction software (horizontal double-arrow lines) [1]. Predicted exons correspond to the chromosomal location of the novel exons −2, −3 and −4 identified in the 5′RACE experiments. Three independent computer algorithms localised putative promoters (dashed horizontal double-arrow lines) in a CpG island 70–72 kb upstream of Ex +1. (TIF) [file pone.0051650.s001.tif]

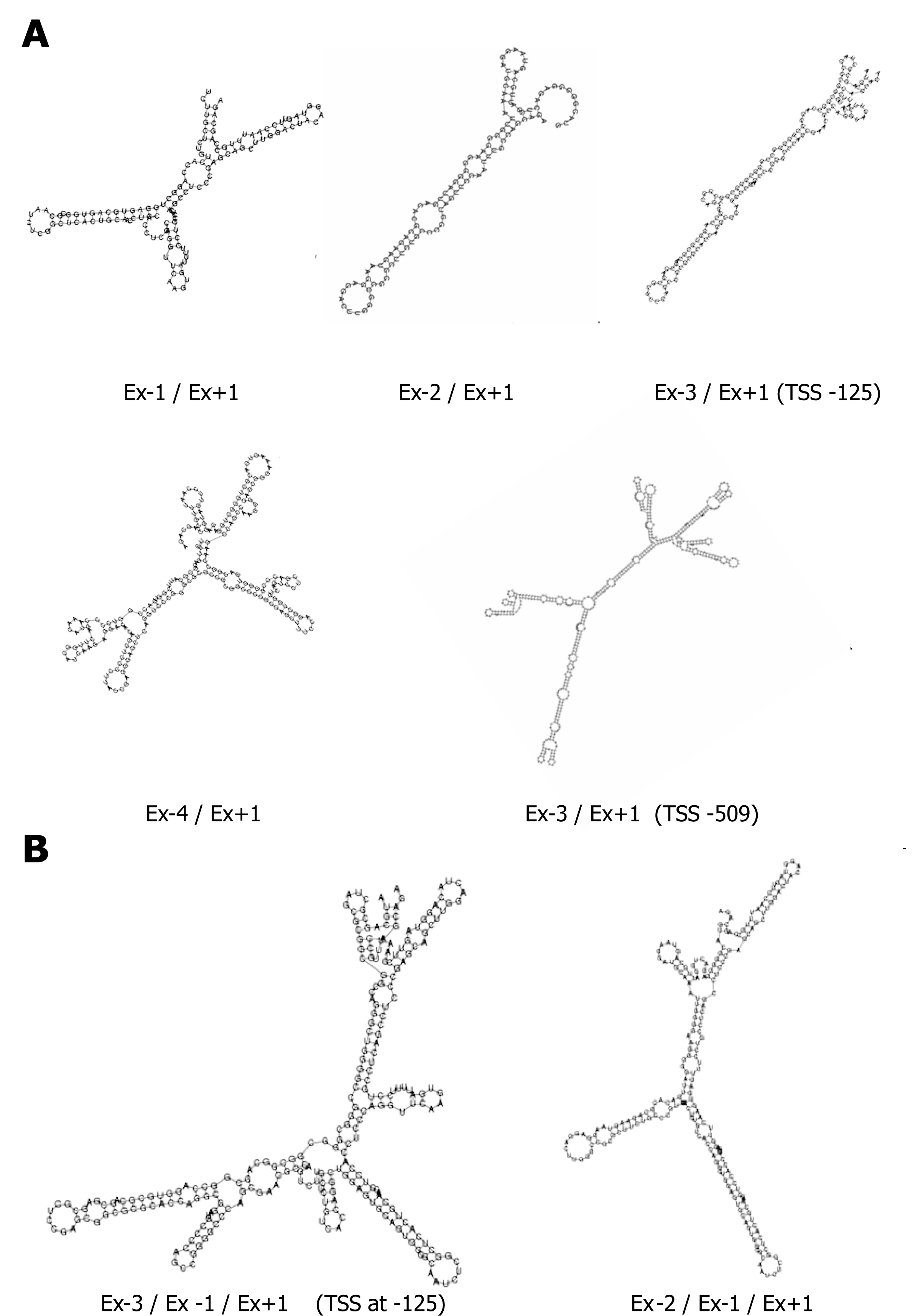

Supplement: Figure S2 — Putative secondary structures of ATP8B1 5′UTR isoforms depicted in Fig. 7 of the main text. Drawings of the minimum free energy (MFE) structures for each splicing variant of ATP8B1 5′UTR suggest their possible different role in regulation of gene expression. One of the regulatory mechanism, formation of stable secondary structures, was shown to impede the progress of the scanning ribosome [2]. Such scanning is influenced by the size and the position of the secondary structure(s) towards the 5′cap of the mRNA species: that is, an alternative transcript with a shorter version of the 5′UTR is frequently translated more efficiently than the one with a longer 5′ region [3], [4], [5]. Likewise, a stem-loop structure located a considerable distance from the 5′cap will require a higher free energy compared to one situated closer to it to affect the access of a pre-initiation complex to the mRNA. [2], [6]. (TIF) [file pone.0051650.s002.tif]
