# Supplementary material for: ATP8B1 Gene Expression Is Driven by a Housekeeping-Like Promoter Independent of Bile Acids and Farnesoid X Receptor
Source: PLoS One. 2012 Dec 10;7(12):e51650. doi: 10.1371/journal.pone.0051650 (PMC3518472; doi:10.1371/journal.pone.0051650)
Supplement: Table S1 — Individually designed TaqMan® MGB probes labelled with Fam and non-fluorescent quencher and primers were generated using Primer Express® Software Version 2.0 (Applied Biosytems), to cover all variants of alternative splicing of the untranslated exons. The abundance of each splicing variant was compared relatively to a non-variable coding region of ATP8B1 represented by Ex+1/+2 boundary. Probe/primer set for Ex+1/+3 boundary (No.14) was used to test the biological significance of transcript excluding protein coding Ex +2, found in EST database (GenBank accession: DR005588.1). All probe sets were designed across exon/exon boundaries to eliminate the possibility of genomic contamination. Primers used in various amplifications are indicated by upper index (a, b, c). The amplification efficiency was tested for each probe/primer set on control templates (obtained by cloning the appropriate cDNA region) using different concentrations of positive and negative controls. As each probe set worked with a slightly different efficiency, the concentration of probes was adjusted for each positive control to reach a cycle threshold (Ct) value difference not greater than 1. (DOC) [file pone.0051650.s003.doc]

**Table S1: Sequence of primers and probes used in qRT-PCR experiments**

| **No.** | **Splicing event** | **Sequence** |
| --- | --- | --- |
| 1. | Ex-4+1 F | GGGCAGCGGGAAAGTGA |
|  | Ex-4+1 R | atgtcgtttctgagtctctttctgtact |
|  | Probe | TGGAgagGTAGTTCCAATT |
| 2. | Ex-4+1_CAG F | AGCGGGAAAGTGACGCTG |
|  | Ex-4+1_CAG R | tctgtactcattctgctggcaaa |
|  | Probe | GAgagcagGTAGTTCCA |
| 3. | Ex-3+1 F | ATGCCCCAGCCGGG^a^ |
|  | Ex-3+1 R | tcattctgctggcaaattgg^b^ |
|  | Probe | CGAACGCcagGTAGT |
| 4. | Ex-3+1_CAG F | ATGCCCCAGCCGGG^a^ |
|  | Ex-3+1_CAG R | tcattctgctggcaaattgg^b^ |
|  | Probe | ACGCcagcagGTAGT |
| 5. | Ex-2+1 F | acttgggccgcgcttc |
|  | Ex-2+1 R | ttctgtactcattctgctggcaa |
|  | Probe | tgcccggtagttccaat |
| 6. | Ex-2+1_CAG F | gacttgggccgcgctt |
|  | Ex-2+1_CAG R | cattctgctggcaaattgga |
|  | Probe | ctgcccgcaggtagt |
| 7. | Ex-3-1 F | ATGCCCCAGCCGGG^a^ |
|  | Ex-3-1 R | aaaatcacttgaacctgggagg |
|  | Probe | AACGCcagTCTTGCT |
| 8. | Ex-2-1 F | AGTAAGGAGGACTTGGGCCG |
|  | Ex-2-1 R | tgcactccagcctggtgac |
|  | Probe | TTCTGCCCGTCTTGCT |
| 9. | Ex-1+1 F | CCCAGGTTCAAGTGATTTTCCT |
|  | Ex-1+1 R | TCTGTACTCATTCTGCTGGCAAA^c^ |
|  | Probe | TTGGACTACAGGTAGTTCCA |
| 10. | Ex-1+1_CAG F | GCCTCCCGAGCAGCTTG |
|  | Ex-1+1_CAG R | TCTGTACTCATTCTGCTGGCAAA^c^ |
|  | Probe | ACTACAGCAGGTAGTTCCA |
| 11. | Ex-3b+1 F | agcgccctcggactcc |
|  | Ex-3b+1 R | tttctgtactcattctgctggca |
|  | Probe | cgcacaggtagttcca |
| 12. | Ex-2b+1 F | gatgtttggagatcacccgg |
|  | Ex-2b+1 F | tttctgtactcattctgctggca |
|  | Probe | cacacggtagttccaatt |
| 13. | Ex+1+2 F | AGAACCGGGAGCCATTCAG |
|  | Ex+1+2 R | tggtacttgcgatcgtttgct |
|  | Probe | AAAAGAATGTACATGGCAAGTC |
| 14. | Ex+1+3 F | agcagaggagaaccgggag |
|  | Ex+1+3 R | aaggtaaatgcgttgtacttgtatgttt |
|  | Probe | cattcagaaaagaataatgcaatt |
