# Supplementary material for: ATP8B1 Gene Expression Is Driven by a Housekeeping-Like Promoter Independent of Bile Acids and Farnesoid X Receptor
Source: PLoS One. 2012 Dec 10;7(12):e51650. doi: 10.1371/journal.pone.0051650 (PMC3518472; doi:10.1371/journal.pone.0051650)
Supplement: Text S1 — Supporting references. (DOC) [file pone.0051650.s004.doc]

**Supplementary references**

1. Davuluri RV, Grosse I, Zhang MQ (2001) Computational identification of promoters and first exons in the human genome. Nat Genet 29: 412-417.

2. Kozak M (1989) Circumstances and mechanisms of inhibition of translation by secondary structure in eucaryotic mRNAs. Mol Cell Biol 9: 5134-5142.

3. Arrick BA, Grendell RL, Griffin LA (1994) Enhanced translational efficiency of a novel transforming growth factor beta 3 mRNA in human breast cancer cells. Mol Cell Biol 14: 619-628.

4. Pickering BM, Willis AE (2005) The implications of structured 5' untranslated regions on translation and disease. Semin Cell Dev Biol 16: 39-47.

5. Hughes TA (2006) Regulation of gene expression by alternative untranslated regions. Trends Genet 22: 119-122.

6. Gray NK, Hentze MW (1994) Regulation of protein synthesis by mRNA structure. Mol Biol Rep 19: 195-200.
